# Docking-based long timescale simulation of cell-size protein systems at atomic resolution

**DOI:** 10.1073/pnas.2210249119

**Published:** 2022-10-03

**Authors:** Ilya A. Vakser, Sergei Grudinin, Nathan W. Jenkins, Petras J. Kundrotas, Eric J. Deeds

**Affiliations:** ^a^Computational Biology Program, The University of Kansas, Lawrence, KS;; ^b^Department of Molecular Biosciences, The University of Kansas, Lawrence, KS;; ^c^University of Grenoble Alpes, CNRS, Grenoble INP, LJK, Grenoble, France;; ^d^Department of Integrative Biology and Physiology, Institute for Quantitative and Computational Biosciences, University of California, Los Angeles, CA

**Keywords:** protein recognition, protein crowding, energy landscape, protein interaction

## Abstract

Computational methodologies are increasingly addressing modeling of the whole cell at the molecular level. Proteins and their interactions are the key component of cellular processes. Techniques for modeling protein interactions, thus far, have included protein docking and molecular simulation. The latter approaches account for the dynamics of the interactions but are relatively slow, if carried out at all-atom resolution, or are significantly coarse grained. Protein docking algorithms are far more efficient in sampling spatial coordinates. However, they do not account for the kinetics of the association (i.e., they do not involve the time coordinate). Our proof-of-concept study bridges the two modeling approaches, developing an approach that can reach unprecedented simulation timescales at all-atom resolution. The global intermolecular energy landscape of a large system of proteins was mapped by the pairwise fast Fourier transform docking and sampled in space and time by Monte Carlo simulations. The simulation protocol was parametrized on existing data and validated on a number of observations from experiments and molecular dynamics simulations. The simulation protocol performed consistently across very different systems of proteins at different protein concentrations. It recapitulated data on the previously observed protein diffusion rates and aggregation. The speed of calculation allows reaching second-long trajectories of protein systems that approach the size of the cells, at atomic resolution.

## Significance

Advances in computational modeling have led to an increasing focus on larger biomolecular systems, up to the level of a cell. Protein interactions are a central component of cellular processes. Techniques for modeling protein interactions have been divided between two fields: protein docking (predicting the static structures of protein complexes) and molecular simulation (modeling the dynamics of protein association, for relatively short simulation times at atomic resolution). Our study combined the two approaches to reach very long simulation times. The study makes the model more adequate to the real cells, to explore cellular processes at atomic resolution to better understand molecular mechanisms of life, and to use this knowledge to improve our ability to treat diseases.

Rapid progress in experimental and computational techniques is redrawing the map of molecular and cellular biology, eliminating old boundaries between research fields, and creating opportunities for breakthroughs. In structural biology, AlphaFold has achieved unprecedented near-experimental accuracy in predicting the structure of individual proteins (1) and, at the same time, a similar approach is successfully used in a different research field—protein docking—to predict the structure of protein complexes (2, 3). Techniques for modeling protein interactions (4) thus far have consisted of two major categories: (1) protein docking (5), such as the fast Fourier transform (FFT) algorithm (which, in short computing times, performs full systematic searches through translational and rotational degrees of freedom) (6), which can be combined with approaches modeling large conformational changes (7–9); and (2) molecular simulations, such as molecular dynamics (MD) or Brownian dynamics (BD) (10). Borrowing from the 4-dimensional (4D) space-time continuum terminology, protein docking has been restricted to sampling of the intermolecular energy landscape at atomic resolution in the 3D space component only, whereas atomic resolution molecular simulation protocols sample the entire 4D landscape, albeit, due to the high computational cost, for short timescales only. Simulation approaches have been applied before, across the fields, to the protein docking problem, broadly for the refinement of the docking global search predictions (9, 11), with more advanced approaches addressing the global docking search itself (12–14). Our study puts forward the reverse across-the-fields application of the docking techniques to the dynamics of the protein interactions.

The great accomplishments in structure prediction based on deep learning do not solve the protein docking problem. This problem, traditionally thought of as a 3D problem, simply requires adding the missing time coordinate from the docking space-time continuum. Refocusing docking from the problem of finding the unique global minimum solution to sampling the enormous multitude of transient interactions (15, 16) dominating the crowded cellular environment allows propagating protein interactions in time. Such propagation can take full advantage of the vast amount of powerful and efficient methodologies accumulated in the protein docking field (5). Thus, it opens extraordinary new opportunities in structural modeling of the biomolecular mechanisms, allowing modeling of larger systems, at longer timescales, all based on the inherent-to-docking atomic resolution.

In the context of the spectacular advances in experimental and computational structural biology, structure-based modeling of protein interactions in the living cell is becoming more central than ever before (17–19). Traditional simulation protocols (e.g., MD, BD) are either relatively slow, if carried out at the all-atom representation (20), or significantly coarse grained, with one particle representing a protein (21). Thus, there are only a few examples of structure-based simulations at the scale of the whole cell (18, 20, 22). Cell modeling is important for a variety of reasons, including integration of data into a unified representation of knowledge about an organism, prediction of multinetwork phenotypes, filling the gaps in our knowledge of cellular processes, and development of our ability to modulate them (17, 23–25). Early approaches to cell modeling represented proteins by hard spheres (21, 26). BD simulations of a part of the *Escherichia coli* cytoplasm were run for 20 μs in rigid-body all-atom representation (27), coarse grained in a subsequent study (28). All-atom MD simulations of bacterial cytoplasm were run for 100 ns (29). Since then, the all-atom MD simulations of cellular environment have reached the microsecond timescales (20, 30–32). Modeling also has been used to study the confinement effect and hydrodynamic properties of the crowded environment (33), the physical limits of cells (34), and packing of the cellular environment (35, 36). The FFT approach was used to study protein folding and binding in the crowded environment (37, 38) and in the free-energy calculations (39).

It has been commonly accepted that mesoscopic particles, such as proteins, in simple solvents can be described with Brownian diffusion. However, this description fails dramatically with molecules in complex biological media, such as the cellular environment (40, 41). While theoretical models can, in principle, explain some of these effects, their applicability requires a priori knowledge of the molecular organization of crowding particles in time and space (42). Thus, simulation techniques, such as MD or BD, are currently the only computational way to access dynamical characteristics of cellular environments. MD simulations are usually restricted to very short timescales. BD simulations allow access to much longer times, but require careful mesoscopic parameterization, for example, with diffusion constants. An alternative to these simulation methods is Monte Carlo (MC) protocols, which allow computing kinetic parameters, such as diffusion coefficients. It requires only computation of the potential energy of the system at each time step. The MC estimate of the self-diffusion coefficient in the continuous move case is in good agreement with the BD simulations (43).

Rigorous experimental tests of the predictions from cell simulations have remained elusive. They have focused almost exclusively on validating predictions of the diffusion coefficient of a protein in a crowded cellular environment by measurements of fluorescent protein diffusion in cells (17, 29). These results showed that effects such as transient interactions and excluded volume significantly decrease the rate of diffusion of proteins in cells (17). Rapidly evolving experimental techniques, such as cryoelectron tomography (44) and high-resolution cryoelectron microscopy (45), time-resolved macromolecular crystallography (46), X-ray photon correlation spectroscopy (47), in-cell NMR spectroscopy (48), and cross-linking mass spectrometry (49, 50) will provide new data on protein diffusion and dynamics of protein association in the crowded cellular environment, including intermediate states and assembly patterns of the protein systems, which can be used for experimental validation of the modeling.

Our proof-of-concept study linked FFT-accelerated systematic docking with the MC simulations, allowing the propagation of large protein systems in time with great computational efficiency. The approach was validated on experimental and computational observations from prior studies and is capable of reaching second-long simulations of the cellular environment at all-atom resolution.

## Materials and Methods

### Modeling Paradigm.

Our approach was to dramatically speed up the sampling of the intermolecular energy landscape by skipping the low-probability (high-energy) states, focusing only on the set of high-probability (low-energy) states corresponding to the energy minima. The “minima hopping” paradigm has been widely used since the early days of molecular modeling for the sampling of the energy landscapes of biomolecules, such as conformational analysis of biopolymers (51), rotamer libraries (52), and refinement of protein–protein interfaces (53), providing extraordinary savings of computing time by avoiding travel in low-probability areas of the landscape. Markov state models have been used to study protein folding, dynamics (54), and association (55) by representing the energy landscape by a set of the energy minima and the probabilities of transition between them. In this study we use a similar idea, namely a Markov state MC approach to sampling transitions between low-energy states, to perform very long trajectory simulations of large systems of proteins at atomic resolution.

### Molecular Systems.

Simulations were performed on three different sets of proteins. To determine the volume fraction of the system, for each protein, the volume was calculated by the 3V server (3vee.molmovdb.org) (56).

**Fig. 1.**
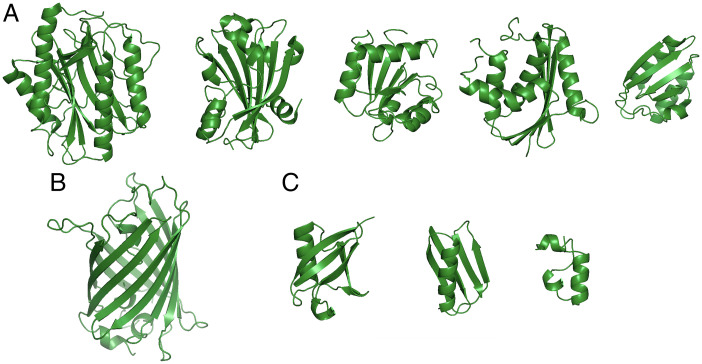
Molecular systems used in the study. (*A*) Five arbitrarily selected globular proteins of average size to represent a typical crowded cellular environment (PDB: 1mat, 1g81, 3chy, 1jxb, and 1cm2). (*B*) GFP (1ema). (*C*) Three small proteins: ubiquitin (1ubq), G protein B subunit (1pga), and villin (1vii). Molecular images were obtained using PyMOL version 2.5 (Schrödinger).

*Set 1*. Five arbitrarily selected globular proteins of average size to represent a “typical” crowded cellular environment (hereafter called “5 mix” set; Fig. 1*A* and *SI Appendix*, Table S1).
*Set 2*. Set 1 plus green fluorescent protein (“GFP + 5 mix” set; Fig. 1*B* and *SI Appendix*, Table S1) for comparison with the experimental data on GFP diffusion.
*Set 3*. Three small proteins (“3 mix” set; Fig. 1*C* and *SI Appendix*, Table S1) from Feig and coworkers (22) representing the nonmembrane part of that study: ubiquitin, G protein B subunit, and villin.

### Generation of the Initial State.

For the starting point of the simulation, the proteins were placed on a cubical grid of a preset size, with the step of the grid calculated according to the desired protein volume fraction. In this study, we used a 500 × 500 × 500-Å^3^ grid (the linear dimension approximately half of that of the smallest cell—*Mycoplasma genitalium*) with periodic boundary conditions. Each protein had an equal share of copies (e.g., in the 5 mix set of the 5-proteins mixture, each protein had a 1 in 5 share of copies). The total number of protein copies and the step of the grid were calculated according to the preset protein volume fraction *V*. In this study, we used a range of volume fraction values, from *V* = 0.10 to close to physiological *V* = 0.30. *SI Appendix*, Table S2 shows the total number of molecular copies corresponding to each volume fraction.

The proteins were placed in a random order. They were randomly rotated and translated within half of the grid step interval. No collision check was applied at this stage since the collisions were eliminated at the start of the simulation. *SI Appendix*, Fig. S1 shows a fragment of the initial state of the system before the start of the simulation.

### Simulation Protocol.

An MC procedure was developed to simulate the cellular environment with proteins in rigid-body approximation, using an all-atom representation. The procedure by design is based on proteins transitioning between different protein–protein associations. Thus, our approach applies to crowded protein environments only, in which proteins encounter each other in close proximity and monomeric states (the absence of all protein–protein interactions, including transient) are uncommon. The energy landscape of the system is represented by our GRAMM FFT docking (6) scores/energies, based on the step function approximation of the Lennard-Jones potential (57). In this representation, the docking poses (including the multiplicity of transient encounters) correspond to negative energy values, and the monomeric states (i.e., the barriers between the minima) have energy zero.

The position of each protein is described with the 3 × 3 rotation matrix and the translation vector relative to the origin of the coordinate system. Protein–protein docking poses are systematically precomputed for all of the rotations and translations of each protein, relative to all of the other proteins in the system by GRAMM docking, unscored and unrefined, at intermediate resolution, previously optimized for the docking of unbound proteins (58) (grid step 3.5 Å, repulsion 9.0, and rotation interval 10°). For proteins A and B, both docking combinations A-B (A is the ligand and B is the receptor) and B-A (B, ligand; A, receptor) are precalculated. Thus, for example, for the 5 mix set the number of precalculated docking outputs is 25 (5 × 5). If A is the moving molecule (ligand), its new putative energy is taken from the A-B docking (and vice versa).

The docking results are stored on 6D grids (3 translations and 3 rotations), accessed during the MC runs. The MC move is initiated by a random selection of a protein (“ligand”) considered for a move to proteins (“receptors”) within a certain neighborhood (described below) from the current position of the ligand. The receptor to move to is selected randomly among all of the neighborhood proteins. Our minima-hopping paradigm, based on the approximation of the Lennard-Jones potential (see above), assumes only the short-distance interactions between the immediate docking partners. The presence of the neighboring proteins not selected for this move is accounted for by the detailed balance condition in the Metropolis acceptance criterion (described below). Once the ligand and the receptor are selected, the move is chosen randomly among the precalculated 30,000 lowest energy-docking matches for that ligand-receptor pair.

The simulation step is completed when all of the proteins have attempted to move. Once the ligand moves, the energy (GRAMM docking score) of the new match is added to the energy of the ligand and the energy of the old match it detaches from is subtracted. Correspondingly, the energy of the new receptor adds the energy of that new match, and the energy from the old receptor (the one the ligand is detaching from) subtracts the energy of the detaching docking match.

**Fig. 2.**
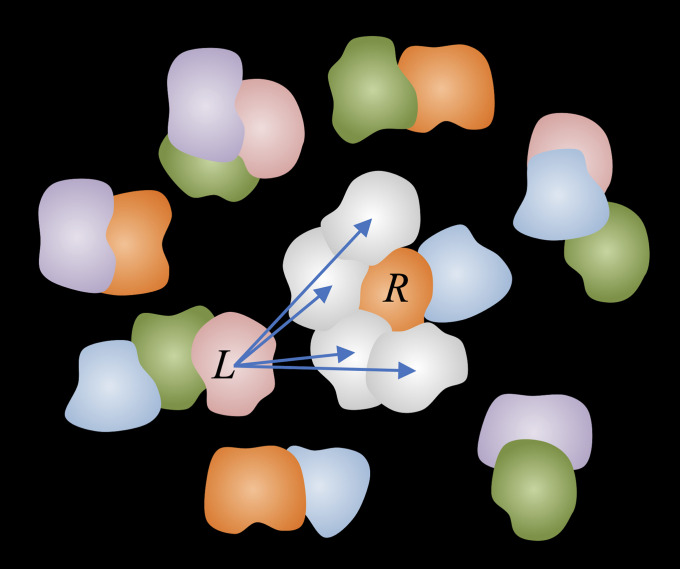
The simulation move set. Docking results are precalculated and stored on 6D grids, accessed during the MC runs. The move set includes a move of one protein (L, ligand) at a time to a putative docking match with another protein (R, receptor) in the vicinity of the ligand. The energies of the states are set according to the docking scores. The move is accepted or rejected based on the Metropolis criterion (detailed balance condition).

The move is accepted or rejected based on the Metropolis acceptance criterion (detailed balance condition). Ligands (L) are allowed to move to the neighboring receptors (R) only (randomly selected among all neighboring proteins), defined as those within the distance between R and L geometric centers less than the sum of the R and L radii, plus 50 Å, to accommodate binding to the first layer of receptors in the crowded environment. Collision check is performed for each attempted move according to the C^α^-C^α^ minimal distance of 8 Å. The moves resulting in collision are rejected. Fig. 2 illustrates the general principle of the move set. Periodic boundary conditions are introduced. Temperature is a parameter to be adjusted for an adequate acceptance rate.

The detailed balance condition for the system was implemented. The probability *P_ij_* of move from step *i* to step *j* had to be the same as *P_ji_* from *j* to *i*. Accordingly, the Metropolis criterion was normalized (59) as

[1] $$P_{ij}=\min\left\{1,\;\exp\left[-\left(E_{j}-E_{i}\right)/T\right]\times N_{i}/N_{j}\right\},$$

where *N_m_* is the numbers of possible moves (receptors to move to; *SI Appendix*, Fig. S2) from state *m* with probability to be selected 1/*N_m_*; *E_m_* is the energy of state *m*; and *T* is the temperature (a scaling factor).

As noted above, in our system, the monomeric states have energy zero, and all of the minima have negative energy values. Our model assumes no additional barriers between states *i* and *j*. We also assume the same curvature of the potential wells of each state. Thus, in the Kramers’ (or Arrhenius) rate equation, which for our system can be written as

[2] $$k=A\cdot P_{ij},$$

where *k* is the rate constant and *P_ij_* is the energy and temperature-based probability of move from step *i* to step *j* (Eq. **1**), the prefactor *A* is the same for all transitions. Thus, our scheme differs from the kinetic MC, because the transition rates are computed on the fly at each step and are proportional (with the constant *A*) to the acceptance probability of a new state.

The observed parameters of the simulation (per simulation step) were potential energy *E*, the average energy of a molecule (the sum of all molecules’ energies—GRAMM docking scores—divided by the number of molecules); the shift (the average length of a molecule’s move per simulation step); the MSD (the average mean square deviation of a molecule’s geometric center after unwrapping coordinates from the periodic boundary conditions); acceptance rate (percentage of accepted moves); and the aggregation number *N_c_* (the average number of proteins in an aggregate/oligomer formed by docked proteins). To allow off-the-grid relaxation of the system, the reference position for MSD calculation was set at step 100. Diffusion rates *D_t_* were calculated from the slope of MSD according to the Einstein relationship *D_t_* = MSD(*t*)/6*t*, where *t* is the lag time.

## Results and Discussion

### Temperature.

**Fig. 3.**
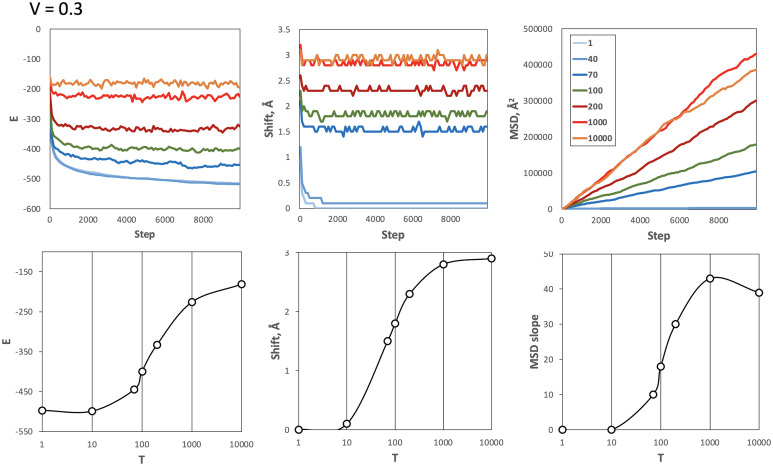
Simulations of the 5 mix set at physiological volume fraction and a range of temperatures. The volume fraction *V* was set to close to physiological 0.3 value. The top panels show the energy *E*, shift, and MSD versus simulation steps. MSD was calculated as the average for 1mat proteins. The temperatures *T* = 1 to 10,000 are shown by different colors. The data on the plots were smoothed by a 100-step averaging sliding window. At low temperatures, the system is frozen (little or no movement of the proteins). At high temperatures, the system is overheated (moves accepted regardless of the energy). The melting curves (the bottom panels in log scale) have a clear inflection point at *T* = 100, indicating the optimal temperature at which the system melts (breaks from the freeze) but is not overheated yet.

The results of the simulation on the 5 mix set at the physiological volume fraction (Fig. 3) and lower volume fractions (*SI Appendix*, Fig. S3) showed that at low temperatures, the system is frozen (little to no movement of the proteins). At high temperatures, the system is overheated (moves accepted regardless of the energy). The melting curves (Fig. 3 and *SI Appendix*, Fig. S3) had a clear inflection point at *T* = 100, consistently at all volume fractions, at which the system melts (breaks from the freeze) but is not overheated yet, and thus is likely most representative of the physiological conditions. The value of *T* corresponding to the melting phase transition reflects the docking energy landscape (mapped in GRAMM energy units), as follows from Eq. **1**, namely the energy gap between a few deep minima (frozen system states) and multiple high-energy/transient states (melted system).

Simulation on the 3 mix set, which is a very different system from the 5 mix set (the 3 mix proteins are much smaller than the ones in the 5 mix), yielded virtually identical melting behavior, at all volume fractions, with the same optimal temperature *T* = 100 (*SI Appendix*, Fig. S4). This confirms the robustness of our approach and adds evidence to the validity of our approximation. Accordingly, for the rest of this study, we used *T* = 100 as the temperature of the systems.

### Calibration.

We calibrated the time units of the simulation protocol on the available data from MD simulation of villin at the physiological volume fraction in the nonmembrane system (22). Here, the diffusion coefficient *D_t_* value was determined to be 3.5 Å^2^/ns, which, according to the authors, is three times greater than in the experiment. Our simulation of the villin within the 3 mix protein set at the physiological volume fraction (*SI Appendix*, Fig. S5) allowed us to calibrate the time variable *t* of our system by matching the *D_t_* values calculated as *D_t_* = MSD/6*t* (see *Materials and Methods*) with the MD results, corrected by the above-mentioned factor of 3. Accordingly, one step of our simulation protocol was determined to be 20 ns.

### Validation and Quantitative Characterization of Protein Systems.

The simulation protocol was validated on a number of observable parameters, testing for consistency of the results and correspondence to experimental and modeling studies. Our minima hopping paradigm, which by design allows no intermediate states between the minima (the minima correspond to the protein bound to another protein), assumes close proximity of the minima to one another (i.e., a crowded environment). Thus, our approximation would not hold for dilute systems. However, it allows for an observation of quantitative characteristics at a range of volume fractions. In our study, this range was set from 0.1 to close to physiological 0.3.

#### Melting temperature.

As described above, the melting temperature for very different protein systems—the 5 mix set of average size proteins and the 3 mix set of much smaller proteins—at the full range of volume fractions, from 0.1 to 0.3, is the same. This supports the validity of our approximation and its consistency across different concentrations and size scales of proteins.

#### Diffusion rate in different systems.

Experimental data on the diffusion of GFP in the cytoplasm of *E. coli* (60) puts the GFP diffusion coefficient *D_t_* in the 0.2 to 0.9 Å^2^/ns range. We ran simulations of the GFP with the 5 mix protein set at a physiological volume fraction. The results (*SI Appendix*, Fig. S6) showed that the GFP diffusion rate was 0.3 Å^2^/ns, in excellent agreement with the experiment. It provides another confirmation of the approach validity and consistency across very disparate systems of proteins.

#### Diffusion rate dependence on concentration.

**Fig. 4.**
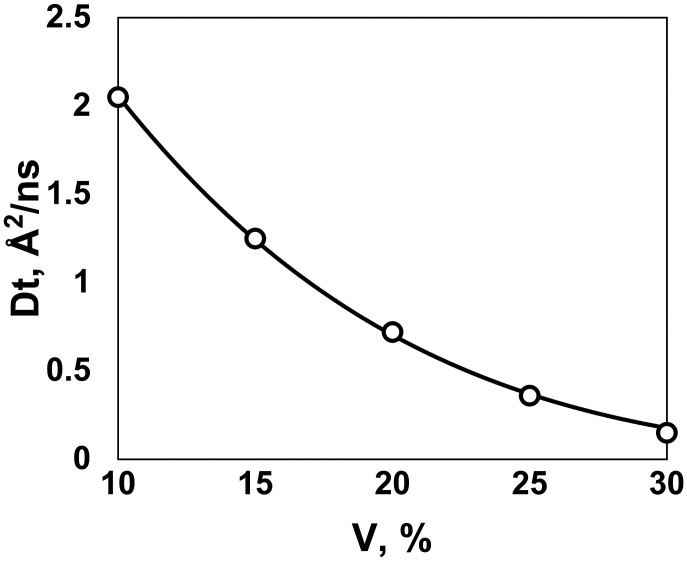
The slowdown of protein diffusion with the increase in protein volume fraction. The diffusion rate *D_t_* was calculated for 1mat proteins in the 5 mix set. The solid line is the data fit by the Cohen-Turnbull expression (61) *D_t_* = *D*_0_ *exp* [−*γ V*/(1 − *V*)], where *D*_0_ is the dilute diffusion rate, and γ is a constant characterizing the slowdown of the diffusion with the increase of the volume fraction *V* (*D*_0_ = 4.9 Å^2^/ns and γ = 7.7 in our simulation).

Simulation in the 5 mix set at different volume fractions showed a pronounced slowdown of the diffusion *D_t_* with the increase in the protein volume fraction *V* in accordance with long-established evidence (20, 22). The data (Fig. 4) are an excellent fit to the Cohen-Turnbull expression (61) *D_t_* = *D*_0_ *exp* [−*γ V*/(1 − *V*)], where *D*_0_ is the dilute diffusion rate and γ is a constant characterizing the slowdown of the diffusion with the increase in the volume fraction (*D*_0_ = 4.9 Å^2^/ns and γ = 7.7 in our simulation). The quantitative scope of this slowdown according to the ratio of the diffusion rates, for our range of volume fractions, is available from the MD simulation for villin as 5.4, from *V* = 0.1 to 0.3 (22). In our simulation of the 3 mix set, that slowdown for villin was 3.4, in good agreement with the MD data.

#### Diffusion rate dependence on size.

**Fig. 5.**
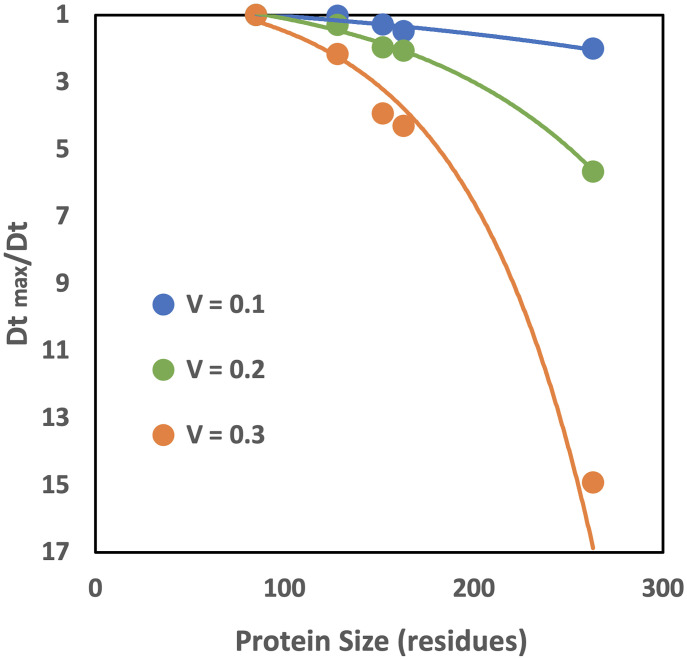
Diffusion rates versus size of proteins. Results obtained on the 5 mix set for the range of volume fractions. The vertical axis shows the slowdown of the diffusion rate relative to the fastest diffusion rate. The slowdown correlates with the size of the protein at all volume fractions.

It is well established by experiment and simulation that larger proteins diffuse at a slower rate (22, 60). Due to the complexity and heterogeneity of the systems, the quantitative estimates of the size versus diffusion correlation vary significantly. Our simulation of sets of small proteins versus those of much larger proteins (see above) showed that the smaller ones diffuse significantly faster. Diffusion of proteins in the same 5 mix set simulation showed clear size versus diffusion rate correlation, at all volume fractions (Fig. 5). A similar trend was observed in the simulation of the 3 mix set (*SI Appendix*, Fig. S7). The rate of the slowdown scales exponentially with the size of the protein defined by the number of residues *N* (*SI Appendix*, Table S3 includes the parameters).

The Einstein-Stokes equation for diffusion of spherical particles predicts that the diffusion rate is inversely proportional to the particle radius. Thus, the slowdown of the diffusion relative to the fastest diffusion rate (*D_t_*_max_/*D_t_*) would have linear dependence on the radius. Our data (*SI Appendix*, Fig. S8), based on the protein size defined by the radius-related metric *R* = *N*^1/3^, show that this dependence is close to linear at lower volume fractions. However, the slowdown rate becomes more pronounced for larger proteins, deviating to exponential at closer to physiological concentrations (60), possibly reflecting the complexity and heterogeneity of the dense protein solutions. Modifying the move set based on the moves acceptance probability (43), which we plan to use in a future study, may provide further insights into the diffusion dependence on protein size at higher volume fractions.

#### Aggregation.

**Fig. 6.**
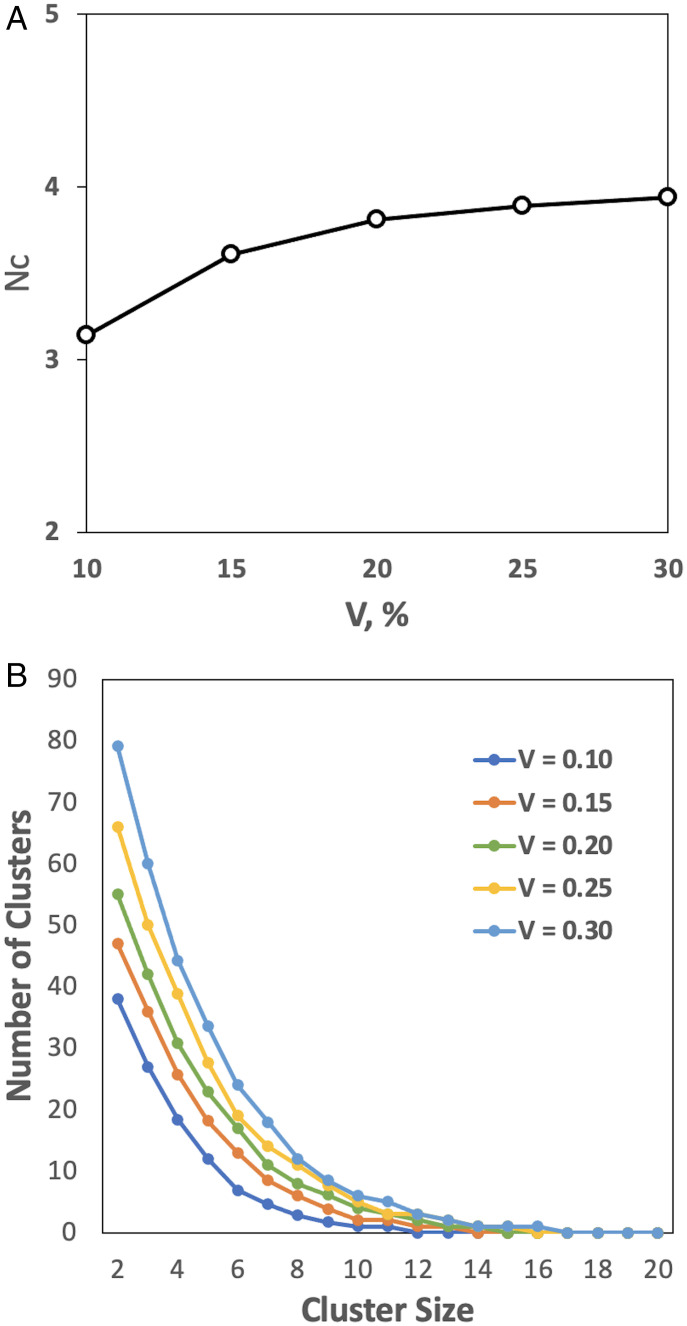
Cluster formation. (*A*) The aggregation number *N_c_* (the average size of protein clusters) across volume fractions *V*. (*B*) Distribution of cluster sizes at different volume fractions. The total number of proteins in the simulation box grows with the increase in the volume fraction (*SI Appendix*, Table S2). Thus, the absolute numbers of clusters at higher volume fractions are larger than those at the lower volume fractions.

Experimental data on the aggregation of proteins (cluster formation) at close to physiological concentrations point to the aggregation number *N_c_* (the average number of proteins in protein assemblies) for lysozyme *N_c_* ≅ 5 (62), and monoclonal antibodies *N_c_* = 4 to 6 (63). Our data obtained on the 5 mix set (Fig. 6*A*), at the physiological volume fraction 0.3, yielded the aggregation number (cluster size) *N_c_* = 3.9, in excellent agreement with these estimates. The results show that the aggregation number does not change much across the whole range of the volume fractions (Fig. 6*A*). This explains the similarity of the energy values *E* per molecule at different volume fractions (Fig. 3 and *SI Appendix*, Fig. S3), since according to our move set, this energy is determined by the number of the protein’s interfaces with other proteins.

The distribution of the cluster sizes (Fig. 6*B*) is in qualitative agreement with the results of the MD simulation in the *N_c_* = 1 to 10 range (22). On average, at each step of the simulation, a small percentage of proteins in our system (4% for *V* = 0.3 and 7% for *V* = 0.1) are monomers (proteins whose partners have moved away and who have not acquired another partner yet, according to our move set).

#### Residence time.

The existing estimates of the proteins’ residence time (the lifetime of a protein pair) vary dramatically among the studies. An experimental study of lysozyme protein solution determined that the protein clusters (complexes) have a lifetime longer than the time required to diffuse over a distance of a monomer diameter (64). Such a distance would correspond to ∼50 steps in our simulation protocol (1 μs). The MD simulation, however, predicted far shorter lifetimes, with most times <20 ns (20). In our simulation, at volume fractions comparable to the ones in the above studies, the protein residence time is ∼570 ns. Thus, our results are between the above experimental and MD estimates.

### Trajectory Length.

**Fig. 7.**
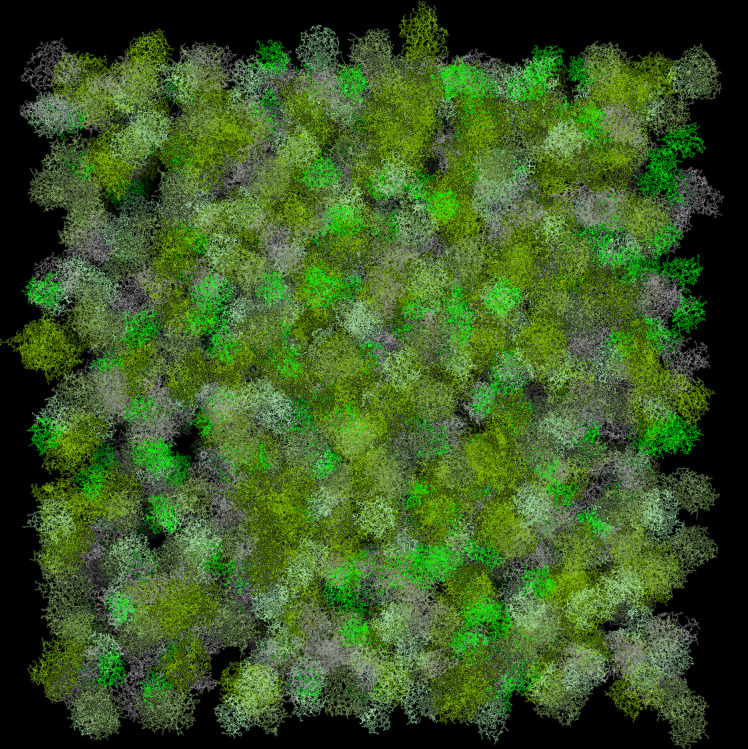
The simulation box. Protein volume fraction is the physiological 0.3. The image was obtained using PyMOL version 2.5 (Schrödinger).

Running the 5 mix protein set in a 500 × 500 × 500-Å^3^ box (the smallest cell is ∼1,000 Å in linear dimension) for 10,000 steps (200 μs) at volume fraction 0.3 (Fig. 7) takes ∼5 h on a 3.1-GHz Intel Core i7 processor (one core). The same calculation at volume fraction 0.1 takes ∼30 min. That puts a 0.3- to 3-s simulation of such system in about 1 year 1 central processing unit-core time frame. Given the all-atom resolution of our approach, this is an extraordinarily long simulation trajectory, which provides an opportunity to explicitly recreate in silico the physiological mechanisms that now are beyond the reach of atomic-resolution simulations.

## Conclusions and Future Directions

Spectacular achievements of the deep learning approaches to protein structure prediction open opportunities for protein docking to refocus from the unique lowest energy states to the enormous multitude of the transient protein interactions that dominate the crowded cellular environment. Taking account of the transient interactions makes it possible to propagate in time the results of static protein docking, thus taking advantage of the powerful and efficient methodologies accumulated in the protein docking field. It opens exciting opportunities in structural modeling of the protein interactions, allowing modeling of larger systems at longer timescales, based on the atomic resolution, which is integral to docking approaches.

Rapid progress in experiment and modeling is leading to the merger of molecular and cellular biology fields. Computational methodologies increasingly address modeling of the whole cell at the molecular level. Whole-cell modeling can provide better understanding of cellular mechanisms and increase our ability to modulate them. The overarching goal, however, is the intellectual challenge of modeling life in silico.

Proteins and their interactions are the key component of cellular processes. Techniques for modeling protein interactions include protein docking and molecular simulation. The latter approaches account for the dynamics of the interactions. However, they are relatively slow, if carried out at the all-atom resolution, or significantly coarse grained (e.g., one particle representing a protein). Protein docking algorithms (e.g., systematic docking by FFT) are far more efficient in sampling the spatial coordinates. However, they do not account for the kinetics of the association (i.e., do not involve the time coordinate). The approach put forward in this study bridges the two modeling techniques. The global intermolecular energy landscape of a large system of proteins was mapped by the pairwise FFT docking and sampled in space and time using MC simulations. The approach is capable of reaching unprecedented simulation timescales at all-atom resolution.

The simulation protocol was parametrized on existing MD data and validated on observations from experiments and MD simulations. The simulation performed consistently across very different systems of proteins at a broad range of concentrations. It recapitulated data on the previously observed protein diffusion rates and aggregation. The speed of calculation allows reaching second-long trajectories of protein systems that approach the size of the cells at atomic resolution.

The long timescale atomic resolution simulations will provide the tool to explore the dynamics of cellular processes in structural detail and address important biological questions based on the molecular mechanisms involving protein association, such as cell signaling pathways and cellular metabolism. These simulations can provide important insights into fundamental biological problems of the specificity of protein interactions, facilitate studies of multinetwork phenotypes, emergent behavior in cellular protein systems, and advance our ability to modulate interaction networks.

This proof-of concept study is obviously just the very beginning of an expansive task of incorporating other types of macromolecules, using more sophisticated force fields that include electrostatics and solvent effects, more accurately accounting for energy barriers, optimizing the move set based on the moves acceptance probability, introducing structural flexibility, adding membrane environment and other cellular components, multiscale modeling, and improving computational efficiency. Nonetheless, our study shows that approaches grounded in protein docking can produce unprecedented dynamic simulations of protein systems at the cellular scale.

## Supplementary Material

Supplementary File

## Data Availability

All of the study data are included in the article and/or supporting information.

## References

[1] J. Jumper , Highly accurate protein structure prediction with AlphaFold. Nature 596, 583–589 (2021). 34265844 10.1038/s41586-021-03819-2 PMC8371605

[2] R. Evans , Protein complex prediction with AlphaFold-Multimer. bioRxiv Preprint (2021). 10.1101/2021.10.04.463034. Accessed 12 December 2021.

[3] M. Baek , Accurate prediction of protein structures and interactions using a three-track neural network. Science 373, 871–876 (2021). 34282049 10.1126/science.abj8754 PMC7612213

[4] D. Murray, D. Petrey, B. Honig, Integrating 3D structural information into systems biology. J. Biol. Chem. 296, 100562 (2021). 33744294 10.1016/j.jbc.2021.100562 PMC8095114

[5] I. A. Vakser, Protein-protein docking: From interaction to interactome. Biophys. J. 107, 1785–1793 (2014). 25418159 10.1016/j.bpj.2014.08.033 PMC4213718

[6] E. Katchalski-Katzir , Molecular surface recognition: Determination of geometric fit between proteins and their ligands by correlation techniques. Proc. Natl. Acad. Sci. U.S.A. 89, 2195–2199 (1992). 1549581 10.1073/pnas.89.6.2195 PMC48623

[7] J. M. Krieger, P. Doruker, A. L. Scott, D. Perahia, I. Bahar, Towards gaining sight of multiscale events: Utilizing network models and normal modes in hybrid methods. Curr. Opin. Struct. Biol. 64, 34–41 (2020). 32622329 10.1016/j.sbi.2020.05.013 PMC7666066

[8] P. M. Khade, A. Kumar, R. L. Jernigan, Characterizing and predicting protein hinges for mechanistic insight. J. Mol. Biol. 432, 508–522 (2020). 31786268 10.1016/j.jmb.2019.11.018 PMC7029793

[9] A. Harmalkar, J. J. Gray, Advances to tackle backbone flexibility in protein docking. Curr. Opin. Struct. Biol. 67, 178–186 (2021). 33360497 10.1016/j.sbi.2020.11.011 PMC9126319

[10] G. A. Huber, J. A. McCammon, Brownian dynamics simulations of biological molecules. Trends Chem. 1, 727–738 (2019). 32309795 10.1016/j.trechm.2019.07.008 PMC7164793

[11] M. F. Lensink, N. Nadzirin, S. Velankar, S. J. Wodak, Modeling protein-protein, protein-peptide, and protein-oligosaccharide complexes: CAPRI 7th edition. Proteins 88, 916–938 (2020). 31886916 10.1002/prot.25870

[12] A. C. Pan , Atomic-level characterization of protein-protein association. Proc. Natl. Acad. Sci. U.S.A. 116, 4244–4249 (2019). 30760596 10.1073/pnas.1815431116 PMC6410769

[13] W. Yu, S. Jo, S. K. Lakkaraju, D. J. Weber, A. D. MacKerell Jr., Exploring protein-protein interactions using the site-identification by ligand competitive saturation methodology. Proteins 87, 289–301 (2019). 30582220 10.1002/prot.25650 PMC6408985

[14] X. Li, I. H. Moal, P. A. Bates, Detection and refinement of encounter complexes for protein-protein docking: Taking account of macromolecular crowding. Proteins 78, 3189–3196 (2010). 20552581 10.1002/prot.22770

[15] D. Kozakov , Encounter complexes and dimensionality reduction in protein-protein association. eLife 3, e01370 (2014). 24714491 10.7554/eLife.01370 PMC3978769

[16] M. I. Freiberger, P. G. Wolynes, D. U. Ferreiro, M. Fuxreiter, Frustration in fuzzy protein complexes leads to interaction versatility. J. Phys. Chem. B 125, 2513–2520 (2021). 33667107 10.1021/acs.jpcb.0c11068 PMC8041309

[17] I. A. Vakser, E. J. Deeds, Computational approaches to macromolecular interactions in the cell. Curr. Opin. Struct. Biol. 55, 59–65 (2019). 30999240 10.1016/j.sbi.2019.03.012 PMC6692245

[18] M. Feig, Y. Sugita, Whole-cell models and simulations in molecular detail. Annu. Rev. Cell Dev. Biol. 35, 191–211 (2019). 31299173 10.1146/annurev-cellbio-100617-062542 PMC6783340

[19] L. Heo, Y. Sugita, M. Feig, Protein assembly and crowding simulations. Curr. Opin. Struct. Biol. 73, 102340 (2022). 35219215 10.1016/j.sbi.2022.102340 PMC8957576

[20] S. von Bülow, M. Siggel, M. Linke, G. Hummer, Dynamic cluster formation determines viscosity and diffusion in dense protein solutions. Proc. Natl. Acad. Sci. U.S.A. 116, 9843–9852 (2019). 31036655 10.1073/pnas.1817564116 PMC6525548

[21] D. J. Bicout, M. J. Field, Stochastic dynamics simulations of macromolecular diffusion in a model of the cytoplasm of *Escherichia coli*. J. Phys. Chem. 100, 2489–2497 (1996).

[22] G. Nawrocki, W. Im, Y. Sugita, M. Feig, Clustering and dynamics of crowded proteins near membranes and their influence on membrane bending. Proc. Natl. Acad. Sci. U.S.A. 116, 24562–24567 (2019). 31740611 10.1073/pnas.1910771116 PMC6900600

[23] J. Carrera, M. W. Covert, Why build whole-cell models? Trends Cell Biol. 25, 719–722 (2015). 26471224 10.1016/j.tcb.2015.09.004 PMC4663153

[24] W. Im , Challenges in structural approaches to cell modeling. J. Mol. Biol. 428, 2943–2964 (2016). 27255863 10.1016/j.jmb.2016.05.024 PMC4976022

[25] Z. R. Thornburg , Fundamental behaviors emerge from simulations of a living minimal cell. Cell 185, 345–360.e28 (2022). 35063075 10.1016/j.cell.2021.12.025 PMC9985924

[26] D. Ridgway , Coarse-grained molecular simulation of diffusion and reaction kinetics in a crowded virtual cytoplasm. Biophys. J. 94, 3748–3759 (2008). 18234819 10.1529/biophysj.107.116053 PMC2367169

[27] S. R. McGuffee, A. H. Elcock, Diffusion, crowding & protein stability in a dynamic molecular model of the bacterial cytoplasm. PLOS Comput. Biol. 6, e1000694 (2010). 20221255 10.1371/journal.pcbi.1000694 PMC2832674

[28] Q. Wang, M. S. Cheung, A physics-based approach of coarse-graining the cytoplasm of Escherichia coli (CGCYTO). Biophys. J. 102, 2353–2361 (2012). 22677389 10.1016/j.bpj.2012.04.010 PMC3353097

[29] I. Yu , Biomolecular interactions modulate macromolecular structure and dynamics in atomistic model of a bacterial cytoplasm. eLife 5, e19274 (2016). 27801646 10.7554/eLife.19274 PMC5089862

[30] M. M. Rickard, Y. Zhang, M. Gruebele, T. V. Pogorelov, In-cell protein-protein contacts: Transient interactions in the crowd. J. Phys. Chem. Lett. 10, 5667–5673 (2019). 31483661 10.1021/acs.jpclett.9b01556

[31] G. Gopan, M. Gruebele, M. Rickard, In-cell protein landscapes: Making the match between theory, simulation and experiment. Curr. Opin. Struct. Biol. 66, 163–169 (2021). 33254078 10.1016/j.sbi.2020.10.013

[32] M. M. Rickard, Y. Zhang, T. V. Pogorelov, M. Gruebele, Crowding, sticking, and partial folding of GTT WW domain in a small cytoplasm model. J. Phys. Chem. B 124, 4732–4740 (2020). 32463238 10.1021/acs.jpcb.0c02536

[33] E. Chow, J. Skolnick, Effects of confinement on models of intracellular macromolecular dynamics. Proc. Natl. Acad. Sci. U.S.A. 112, 14846–14851 (2015). 26627239 10.1073/pnas.1514757112 PMC4672785

[34] K. A. Dill, K. Ghosh, J. D. Schmit, Physical limits of cells and proteomes. Proc. Natl. Acad. Sci. U.S.A. 108, 17876–17882 (2011). 22006304 10.1073/pnas.1114477108 PMC3207669

[35] G. T. Johnson , cellPACK: A virtual mesoscope to model and visualize structural systems biology. Nat. Methods 12, 85–91 (2015). 25437435 10.1038/nmeth.3204 PMC4281296

[36] M. Maritan , Building structural models of a whole Mycoplasma cell. J. Mol. Biol. 434, 167351 (2022). 34774566 10.1016/j.jmb.2021.167351 PMC8752489

[37] S. Qin, H. X. Zhou, An FFT-based method for modeling protein folding and binding under crowding: Benchmarking on ellipsoidal and all-atom crowders. J. Chem. Theory Comput. 9, 4633–4643 (2013). 10.1021/ct4005195 PMC3811151 24187527

[38] S. Qin, H. X. Zhou, Further development of the FFT-based method for atomistic modeling of protein folding and binding under crowding: Optimization of accuracy and speed. J. Chem. Theory Comput. 10, 2824–2835 (2014). 25061446 10.1021/ct5001878 PMC4095916

[39] T. H. Nguyen, H. X. Zhou, D. D. L. Minh, Using the fast Fourier transform in binding free energy calculations. J. Comput. Chem. 39, 621–636 (2018). 29270990 10.1002/jcc.25139 PMC5834390

[40] F. Höfling, T. Franosch, Anomalous transport in the crowded world of biological cells. Rep. Prog. Phys. 76, 046602 (2013). 23481518 10.1088/0034-4885/76/4/046602

[41] J. A. Dix, A. S. Verkman, Crowding effects on diffusion in solutions and cells. Annu. Rev. Biophys. 37, 247–263 (2008). 18573081 10.1146/annurev.biophys.37.032807.125824

[42] C. Cruz, F. Chinesta, G. Regnier, Review on the Brownian dynamics simulation of bead-rod-spring models encountered in computational rheology. Arch. Comput. Methods Eng. 19, 227–259 (2012).

[43] E. Sanz, D. Marenduzzo, Dynamic Monte Carlo versus Brownian dynamics: A comparison for self-diffusion and crystallization in colloidal fluids. J. Chem. Phys. 132, 194102 (2010). 20499946 10.1063/1.3414827

[44] F. J. B. Bäuerlein, W. Baumeister, Towards visual proteomics at high resolution. J. Mol. Biol. 433, 167187 (2021). 34384780 10.1016/j.jmb.2021.167187

[45] S. J. Ziegler, S. J. B. Mallinson, P. C. St John, Y. J. Bomble, Advances in integrative structural biology: Towards understanding protein complexes in their cellular context. Comput. Struct. Biotechnol. J. 19, 214–225 (2020). 33425253 10.1016/j.csbj.2020.11.052 PMC7772369

[46] G. Brändén, R. Neutze, Advances and challenges in time-resolved macromolecular crystallography. Science 373, eaba0954 (2021). 34446579 10.1126/science.aba0954

[47] Y. Chushkin , Probing cage relaxation in concentrated protein solutions by XPCS. arXiv [Preprint] (2022). 10.48550/arXiv.2203.12695. Accessed 16 August 2022. 36563210

[48] M. Gruebele, G. J. Pielak, Dynamical spectroscopy and microscopy of proteins in cells. Curr. Opin. Struct. Biol. 70, 1–7 (2021). 33662744 10.1016/j.sbi.2021.02.001

[49] X. Tang, H. H. Wippel, J. D. Chavez, J. E. Bruce, Crosslinking mass spectrometry: A link between structural biology and systems biology. Protein Sci. 30, 773–784 (2021). 33594738 10.1002/pro.4045 PMC7980526

[50] X. Liu , Driving integrative structural modeling with serial capture affinity purification. Proc. Natl. Acad. Sci. U.S.A. 117, 31861–31870 (2020). 33257578 10.1073/pnas.2007931117 PMC7749342

[51] S. G. Galaktionov, V. M. Tseĭtin, I. A. Vakser, E. V. Prokhorchik, [Amphiphilic properties of angiotensin and its fragments]. Biofizika 33, 556–558 (1988). 3191167

[52] T. Kirys, A. M. Ruvinsky, A. V. Tuzikov, I. A. Vakser, Rotamer libraries and probabilities of transition between rotamers for the side chains in protein-protein binding. Proteins 80, 2089–2098 (2012). 22544766 10.1002/prot.24103 PMC3393779

[53] T. Dauzhenka, P. J. Kundrotas, I. A. Vakser, Computational feasibility of an exhaustive search of side-chain conformations in protein-protein docking. J. Comput. Chem. 39, 2012–2021 (2018). 30226647 10.1002/jcc.25381 PMC6186188

[54] D. Shukla, C. X. Hernández, J. K. Weber, V. S. Pande, Markov state models provide insights into dynamic modulation of protein function. Acc. Chem. Res. 48, 414–422 (2015). 25625937 10.1021/ar5002999 PMC4333613

[55] Z. He, F. Paul, B. Roux, A critical perspective on Markov state model treatments of protein-protein association using coarse-grained simulations. J. Chem. Phys. 154, 084101 (2021). 33639768 10.1063/5.0039144 PMC7902085

[56] N. R. Voss, M. Gerstein, 3V: Cavity, channel and cleft volume calculator and extractor. Nucleic Acids Res. 38, W555-62 (2010). 20478824 10.1093/nar/gkq395 PMC2896178

[57] I. A. Vakser, Long-distance potentials: An approach to the multiple-minima problem in ligand-receptor interaction. Protein Eng. 9, 37–41 (1996). 9053900 10.1093/protein/9.1.37

[58] A. M. Ruvinsky, I. A. Vakser, Chasing funnels on protein-protein energy landscapes at different resolutions. Biophys. J. 95, 2150–2159 (2008). 18515374 10.1529/biophysj.108.132977 PMC2517039

[59] W. K. Hastings, Monte Carlo sampling methods using Markov chains and their applications. Biometrika 57, 97–109 (1970).

[60] A. Nenninger, G. Mastroianni, C. W. Mullineaux, Size dependence of protein diffusion in the cytoplasm of *Escherichia coli*. J. Bacteriol. 192, 4535–4540 (2010). 20581203 10.1128/JB.00284-10 PMC2937421

[61] T. J. O’Leary, Concentration dependence of protein diffusion. Biophys. J. 52, 137–139 (1987). 3607221 10.1016/S0006-3495(87)83199-5 PMC1329994

[62] A. Stradner , Equilibrium cluster formation in concentrated protein solutions and colloids. Nature 432, 492–495 (2004). 15565151 10.1038/nature03109

[63] T. M. Scherer, J. Liu, S. J. Shire, A. P. Minton, Intermolecular interactions of IgG1 monoclonal antibodies at high concentrations characterized by light scattering. J. Phys. Chem. B 114, 12948–12957 (2010). 20849134 10.1021/jp1028646

[64] Y. Liu , Lysozyme protein solution with an intermediate range order structure. J. Phys. Chem. B 115, 7238–7247 (2011). 21114324 10.1021/jp109333c

