# Supplementary material for: Docking-based long timescale simulation of cell-size protein systems at atomic resolution
Source: Proc Natl Acad Sci U S A. 2022 Oct 3;119(41):e2210249119. doi: 10.1073/pnas.2210249119 (PMC9565162; doi:10.1073/pnas.2210249119)
Supplement: Supplementary File [file pnas.2210249119.sapp.pdf]

Supplementary Information for

Docking-based long timescale simulation of cell-size protein systems at atomic resolution

Ilya A. Vakser[1,2] *, Sergei Grudinin[3], Nathan W. Jenkins[1], Petras J. Kundrotas[1], Eric J. Deeds[4]

[1]Computational Biology Program, The University of Kansas, Lawrence, Kansas, USA

[2]Department of Molecular Biosciences, The University of Kansas, Lawrence, Kansas, USA

[3]University of Grenoble Alpes, CNRS, Grenoble INP, LJK, Grenoble, France

[4]Department of Integrative Biology and Physiology, Institute for Quantitative and Computational Biosciences, University of California Los Angeles, California, USA

*Corresponding author:
Ilya A. Vakser
Email: vakser@ku.edu

This PDF file includes:

Figures S1 to S8
Tables S1 to S3

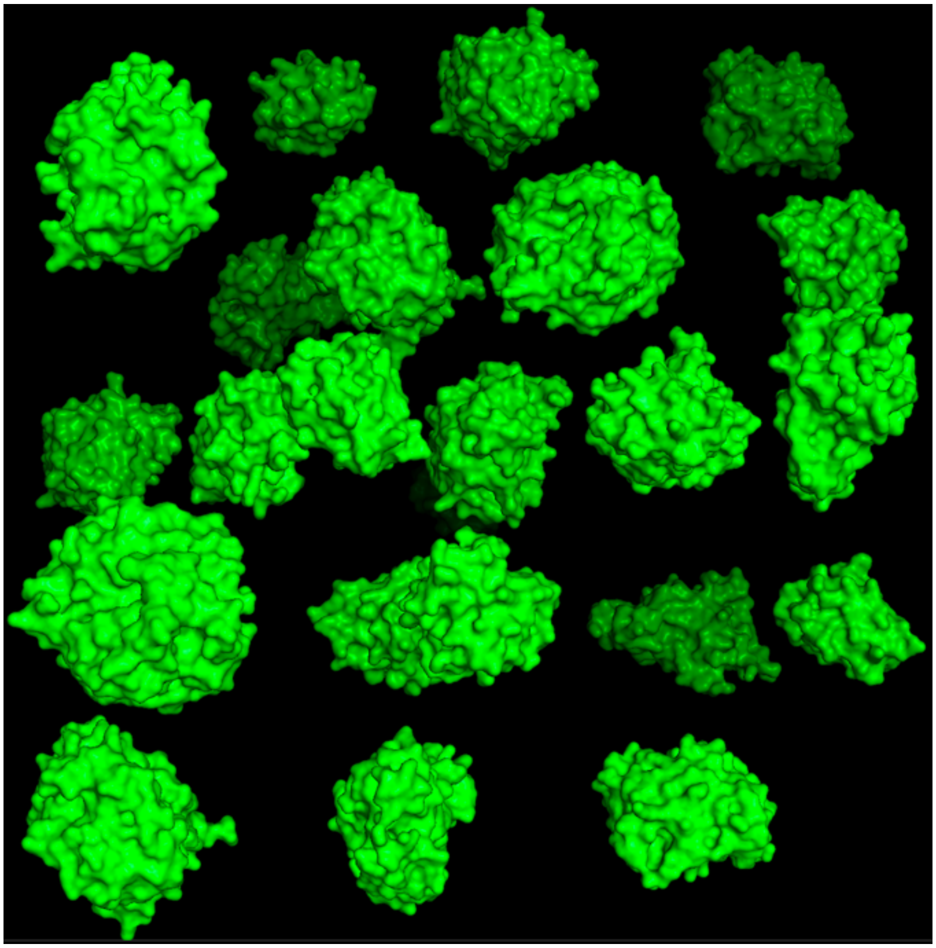

Figure S1. *A fragment of the initial state of the system before the start of simulation*. The volume fraction shown is 0.10. Proteins were placed on a cubical grid in random order, and randomly rotated and translated within half of the grid step. No collision check was applied since the collisions are eliminated at the start of the simulation.

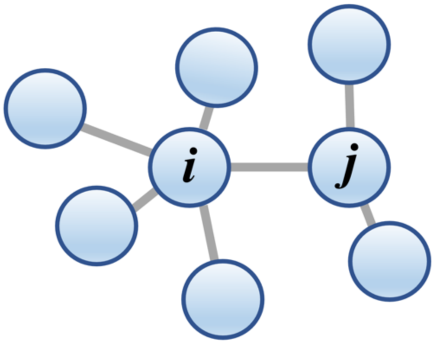

Figure S2. *A possible move set from states i and j.*

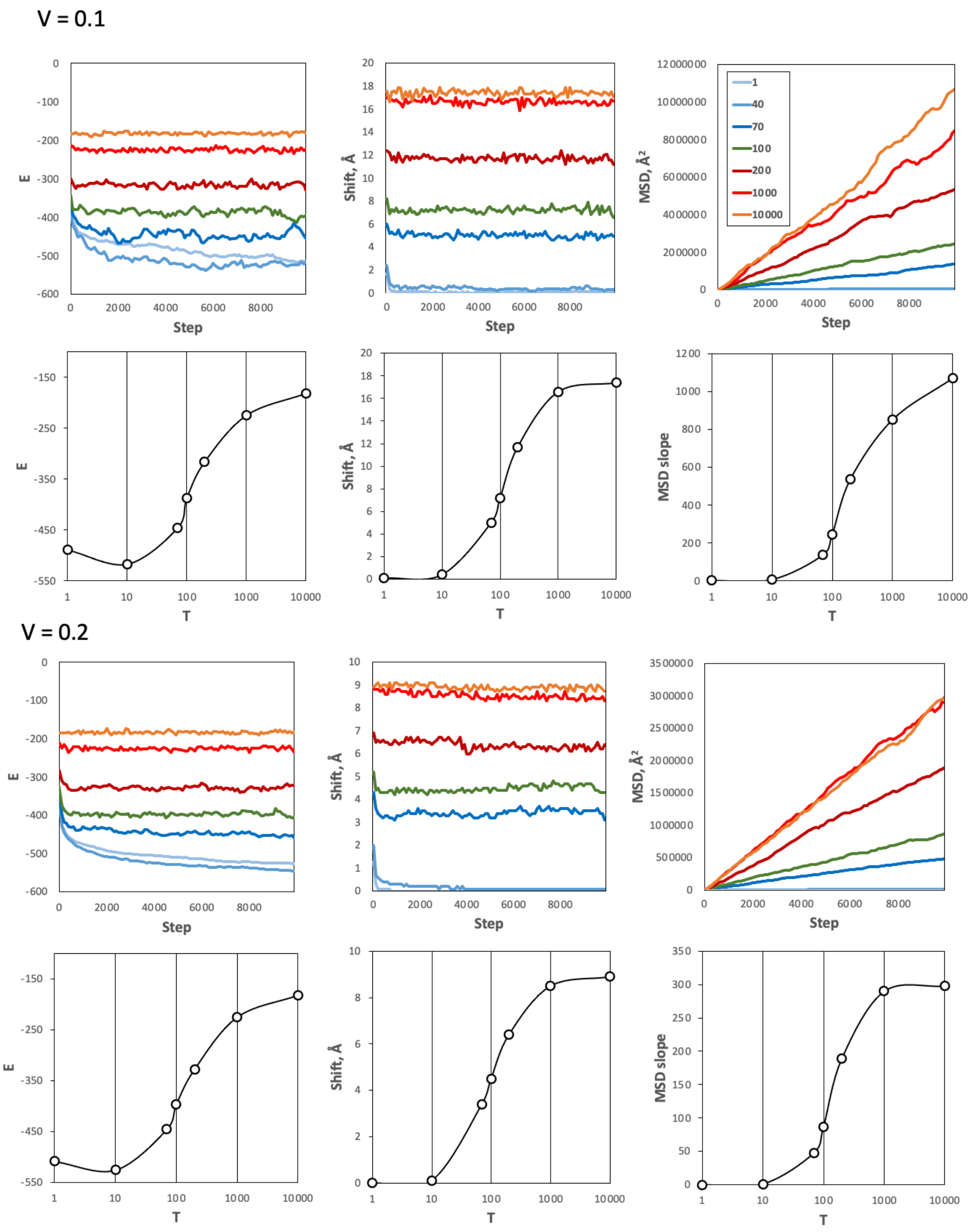

Figure S3. *Simulations of the "5 mix" set at lower than physiological volume fractions and a range of temperatures*. For each volume fraction *V*, the top panels show the energy *E*, shift, and MSD vs. simulation steps. MSD was calculated as the average for 1mat proteins. The temperatures *T* = 1 - 10,000 are shown by different colors. The data on the plots was smoothed by a 100-steps averaging sliding window. At low temperatures, the system is frozen (little or no movement of the proteins). At high temperatures, the system is overheated (moves accepted regardless of the energy). The melting curves (the bottom panels in log scale) have a clear inflection point at *T* = 100 indicating the optimal temperature at which the system melts (breaks from the freeze) but is not overheated yet.

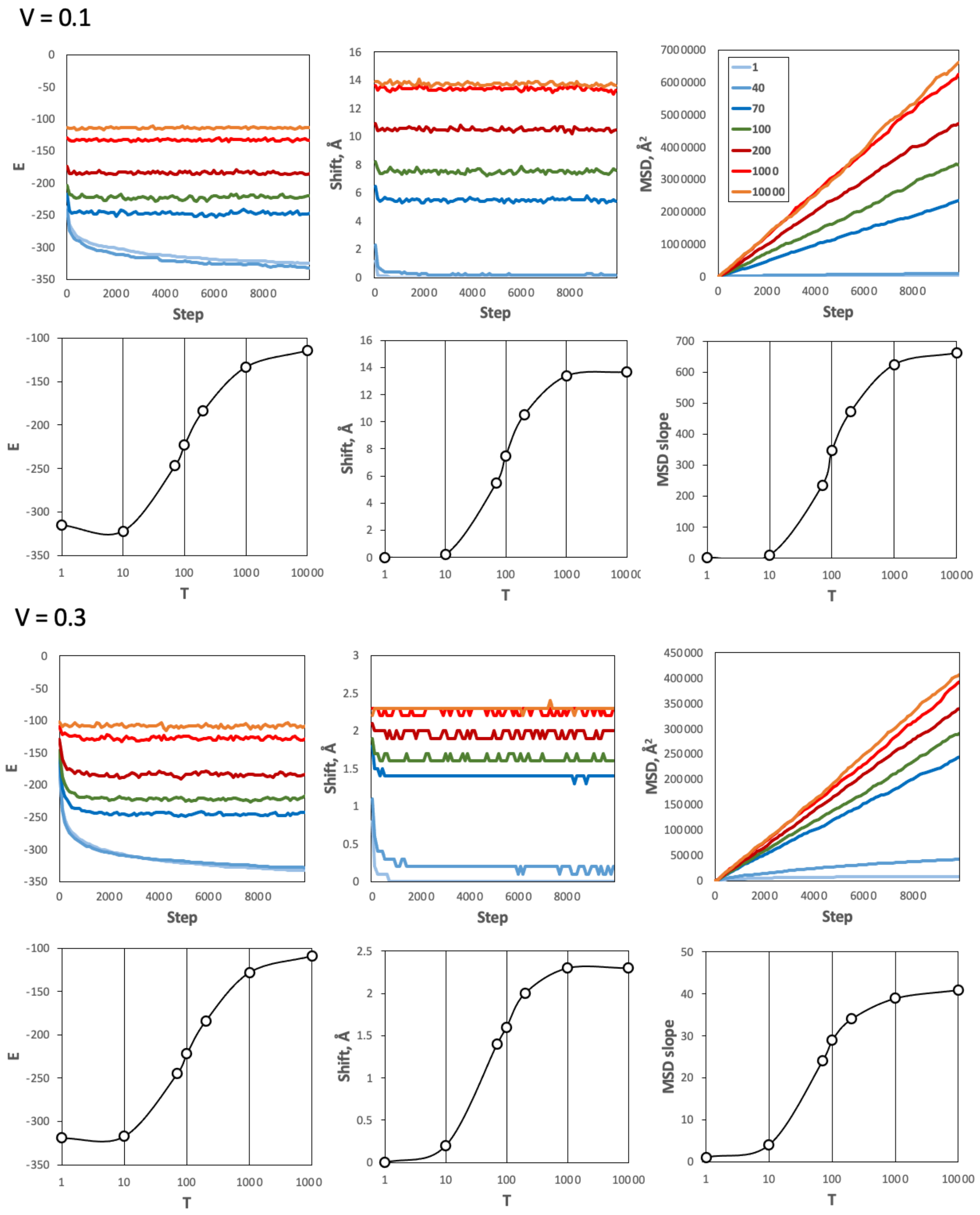

Figure S4. *Simulations of the "3 mix" set at low and physiological volume fractions and a range of temperatures*. For each volume fraction *V*, the top panels show the energy *E*, shift, and MSD vs. simulation steps. MSD was calculated as the average for the ubiquitin (1ubq) proteins. The details of the observable parameters are the same as in Figure S3.

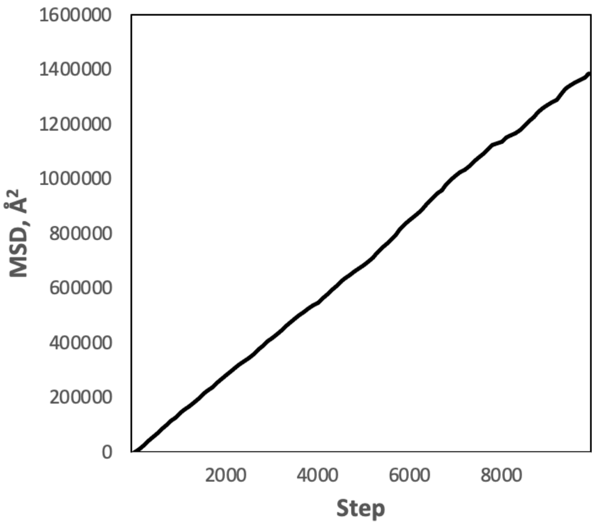

Figure S5. *Simulation of Villin within the "3 mix" protein set*. The simulation was run at T = 100 and V = 0.3 (see text). MSD was calculated as the average for the Villin proteins. The details of the observable parameters are the same as in Figure S3. The system's time variable t was calibrated by matching the D_t value, calculated from the slope of the MSD, as D_t = MSD/6t, with the previously determined D_t values (see text). One step of our simulation protocol was thus determined to be 20 ns.

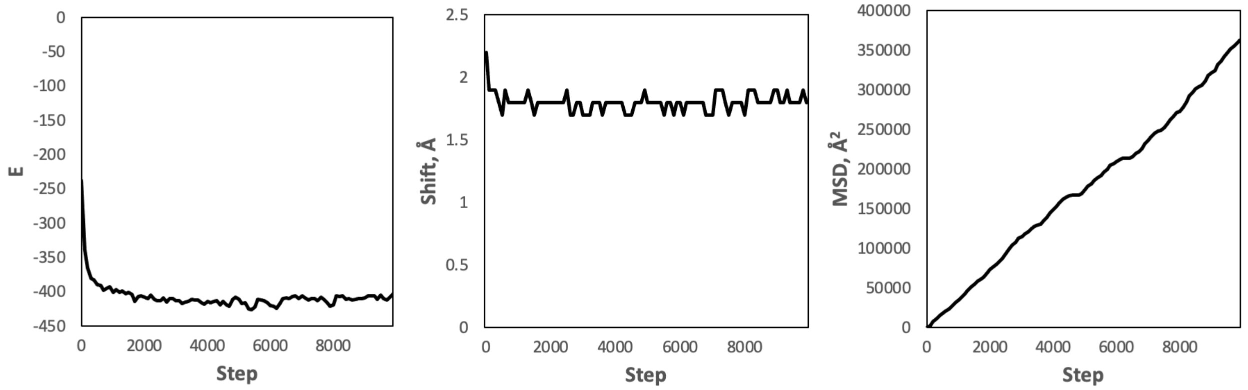

Figure S6. *Simulation of the GFP with the "5 mix" protein set*. The simulation was run at T = 100 and V = 0.3 (see text). MSD was calculated as the average for the GFP proteins. The details of the observable parameters are the same as in Figure S3.

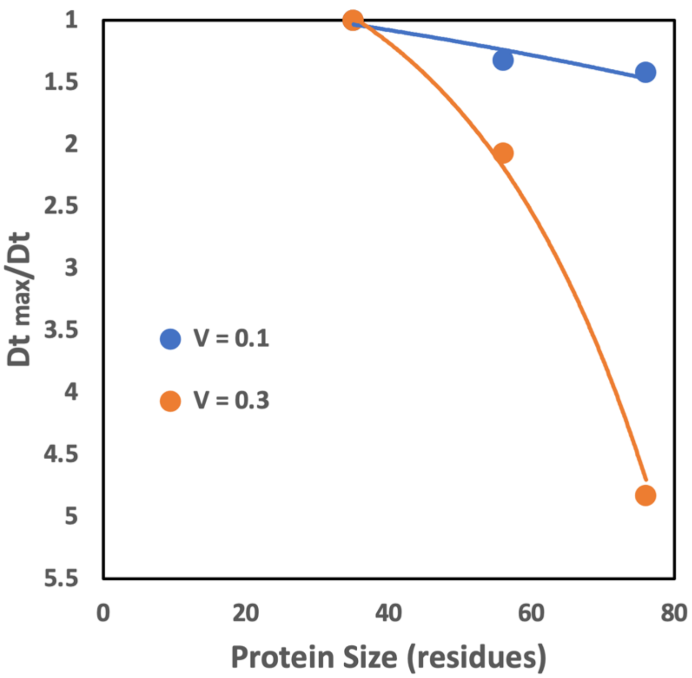

Figure S7. *Diffusion vs. size of proteins*. Results obtained on the "3 mix" set for volume fractions *V* = 0.1 and 0.3. The vertical axis shows the slowdown of the diffusion rate relative to the fastest diffusion rate. The slowdown correlates with the size of the protein at both volume fractions.

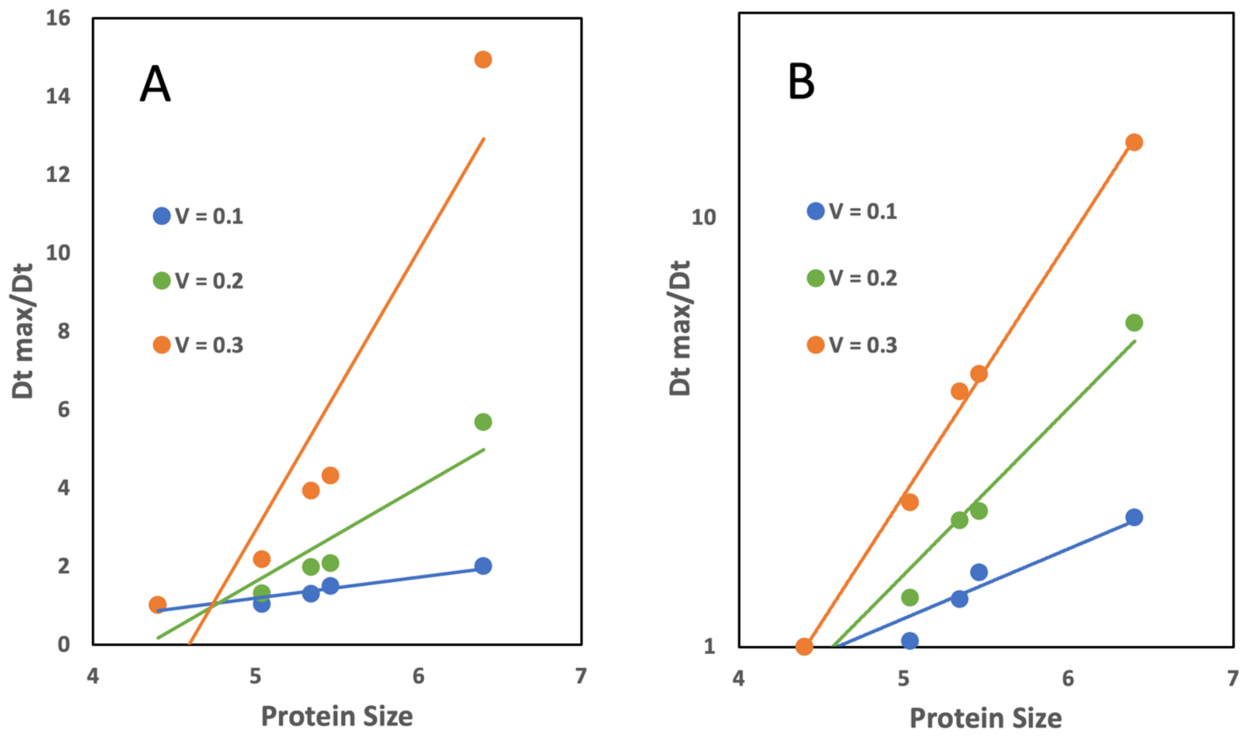

Figure S8. *Diffusion slowdown vs. size of proteins*. The vertical axis shows the slowdown of the diffusion rate relative to the fastest diffusion rate. The size of the proteins is estimated as the radius related metric $R = N^{1/3}$, where N is the number of residues. The data is shown in (A) linear and (B) logarithmic scales. While the size dependence is close to linear at lower volume fractions, it becomes more pronounced, deviating to exponential at closer to physiological concentrations.

Table S1. *Characteristics of the proteins*.

Size	5 mix set					GFP	3 mix set		
	1mat[1]	1g81	3chy	1jxb	1cm2	1ema	1ubq	1pga	1vii
No. of residues	263	163	128	152	85	210	76	56	35
Volume, $Å^3$	40,693	27,823	21,151	25,753	13,720	36,767	12,965	9,019	6,583

[1]PDB codes.

Table S2. *Characteristics of the molecular systems*.

Volume fraction	Number of molecules in simulation system		
	5 mix set	GFP + 5 mix[1]	3 mix set[2]
0.10	460		1,314
0.15	725		
0.20	970		
0.25	1,210		
0.30	1,450	1,356	3,939

[1]GFP + 5 mix set was run only at physiological volume fraction 0.30 at which the experimental data was obtained.

[2]3 mix set was run only at volume fractions 0.1 and 0.3 for which the molecular dynamics data was available.

Table S3. *Parameters of the protein diffusion rate dependence on molecular size*.

Parameters[1]	Volume fraction V		
	0.1	0.2	0.3
A	0.6815	0.4058	0.3329
B	0.0042	0.0100	0.0149

[1]The diffusion slowdown is defined as the ratio of the fastest diffusion rate $D_{t\,\max}$ to the diffusion rate D_t. The slowdown is approximated by $A\ exp(BN)$, where N is the number of residues in the protein.
